# Augmentation index as an early marker of in-hospital mortality in patients with acute ischemic stroke

**DOI:** 10.11604/pamj.2019.34.171.20249

**Published:** 2019-12-02

**Authors:** Athanasia Papazafiropoulou, Eleni-Margarita Tzouganatou, Styliani Papantoniou, Elias Georgopoulos, Andreas Melidonis

**Affiliations:** 1 First Department of Internal Medicine and Diabetes Center, General Hospital "Tzaneio", Piraeus, Greece

**Keywords:** Arterial stiffness, pulse wave velocity, augmentation index, in-hospital mortality, acute ischemic stroke

## To the editors of the Pan African Medical Journal

Stroke is one of the leading causes of death resulting in long term disability worldwide [1]. Outcome of stroke varies and early risk stratification in these patients is essential for their treatment as well as rehabilitation. Arterial stiffness is an established marker of arterial structural and functional alteration [2]. While a growing number of studies have demonstrated its association with stroke [3, 4]. Therefore, the aim of the present study was to estimate the relationship between arterial stiffness, expressed in terms of pulse wave velocity (PWV) and augmentation index (AIx), with stroke severity and outcome. We prospectively studied all patients who were admitted in our Department with acute ischemic stroke between January 2019 and July 2019 (n=94; 36 males, age 79.8 ± 9.1 years). On admission, demographic data and medical history were recorded and a full clinical examination, including laboratory tests, was performed. The severity of stroke was assessed with the National Institutes of Health Stroke Scale (NIHSS) score and the Rankin score. In addition, AIx and PWV were recorded with the Mobil-O-Graph PWA. All data were analyzed using the statistical package SPSS (version 19.0; SPSS, Chicago, IL, USA). In-hospital mortality was 15.6% and mean duration of hospital stay was 6.3 ±4.7 days. Mean value of AIx was 30.6±12.3% and PWV was 12.8±2.1 m/sec. Patients who died during hospitalization had lower systolic blood pressure and higher white blood cells count, higher NIHSS score and Rankin score on admission while there was no difference between AIx and PWV. Multivariate regression analysis, after adjustment for gender, duration of hospital stay, smoking status, history of previous stroke, arterial hypertension, dyslipidemia, NIHSS score and Rankin score, showed that AIx was related only with age (beta=0.49, p=0.03) and in-hospital mortality (beta= -0.43, p=0.04), while there was a trend for association with history of diabetes mellitus (beta= -0.36, p=0.08). No association was found between PWV and the examined variables. The results of the present study showed an inverse association between arterial stiffness, expressed in terms of Aix, and in-hospital mortality while no association between PWV and in-hospital mortality was observed. Similar findings demonstrated a study by Tziomalos et al. where increased AIx was associated with lower risk for in-hospital mortality in patients with acute ischemic stroke [5]. However, the results on the effect of AIx to patient's outcome after stroke are conflicting. Two studies showed no association between AIx and functional outcome at 30 days or with in-hospital outcome [6, 7]. On the other hand, a small study reported better functional outcome at discharge in patients with acute ischemic stroke who had lower Aix [8]. In another study, high AIx was a significant predictor for 3-month mortality in patients with intracerebral hemorrhage [9].

## Conclusion

Decreased Aix and elder age are associated with higher in-hospital mortality in patients with acute ischemic stroke. Further studies are needed in order to clarify the paradoxical effect of arterial stiffness to patient's outcome after acute ischemic stroke.

## Competing interests

The authors declare no competing interests.
